# Postirradiation pseudosclerodermatous panniculitis: A dramatic, delayed, and recalcitrant case of a rare entity

**DOI:** 10.1016/j.jdcr.2025.08.002

**Published:** 2025-08-22

**Authors:** Maria L. Mihailescu, Mitul B. Modi, David C. Reid

**Affiliations:** Department of Dermatology, Rush University Medical Center, Chicago, Illinois

**Keywords:** breast induration, delayed, fat necrosis, foamy histiocytes, lobular panniculitis, postirradiation pseudosclerodermatous panniculitis, radiotherapy, rare

## Introduction

Postirradiation pseudosclerodermatous panniculitis (PIPP) is a rare complication of external beam radiotherapy. Most cases of PIPP present with localized induration, take place within 2 years of radiotherapy, and burn-out spontaneously. Herein, we present a dramatic case of PIPP involving the entire right breast extending to the thoracic muscles with a delayed onset of 27 years after radiotherapy. Our patient’s PIPP continued to progress despite high-dose oral immunosuppressive therapy.

## Case report

An 83-year-old female patient with a history of ductal carcinoma of the right breast treated by lumpectomy and external beam radiation 27 years prior was referred to our dermatology clinic for a 6-month history of dramatic and rapid contraction of the breast with induration. She described asymptomatic “hardening and shrinking” of the circumferential breast extending to the anterior chest wall. Prior to referral, she had been evaluated by her breast surgeon who conducted imaging studies and consecutive core biopsies which were negative for recurrent malignancy. The patient denied breast trauma or cosmetic procedures. She denied fever, weight loss, or other systemic symptoms.

**Fig 1:**
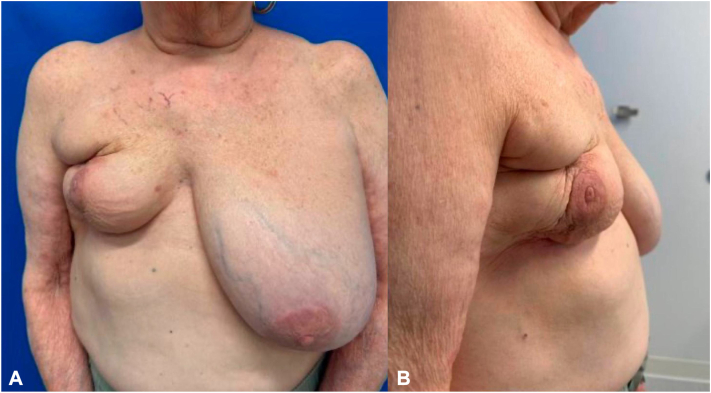
**A,** Marked reduction in right breast size and multiple firm, nodular, indurated, and fixed plaques on the right anterior chest wall **(B)** Plaques extend to the lateral chest wall.

Physical exam revealed marked reduction in right breast size with multiple firm, nodular, and indurated plaques involving the entirety of the right anterior chest wall without any epidermal change (Fig 1). The following laboratory workup was performed and returned negative: QuantiFERON-TB Gold, alpha-1 antitrypsin, lipase, antinuclear antibody panel, antineutrophil cytoplasmic antibodies panel, complement levels, immunoglobulin levels, serum protein electrophoresis, complete blood count, and comprehensive metabolic panel.

**Fig 2:**
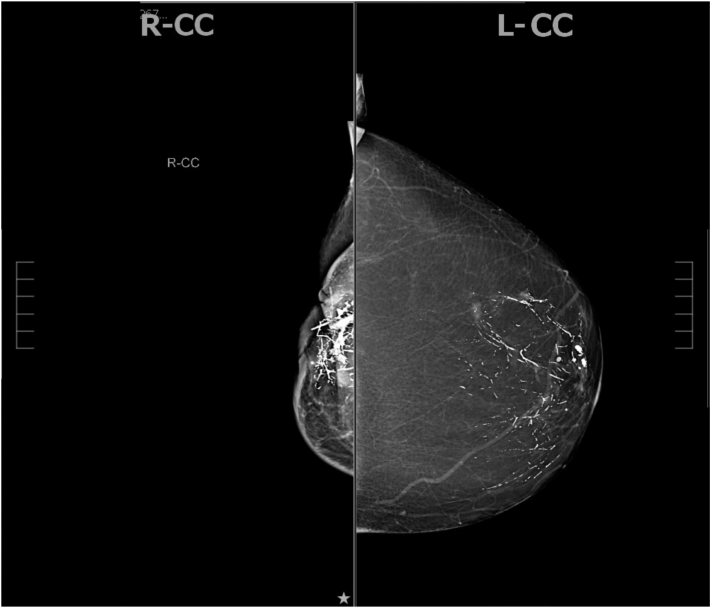
Mammography of the right breast demonstrates an abnormal nonmass enhancement just beneath the dermis extending into the upper outer quadrant measuring 5.1 × 2.9 × 5.4 cm. The left breast appears unremarkable.

Mammography of the right breast revealed an abnormal nonmass enhancement just beneath the dermis of the retroareolar right breast extending into the upper outer quadrant. The total abnormal enhancement measured 5.1 × 2.9 × 5.4 cm (Fig 2).

**Fig 3:**
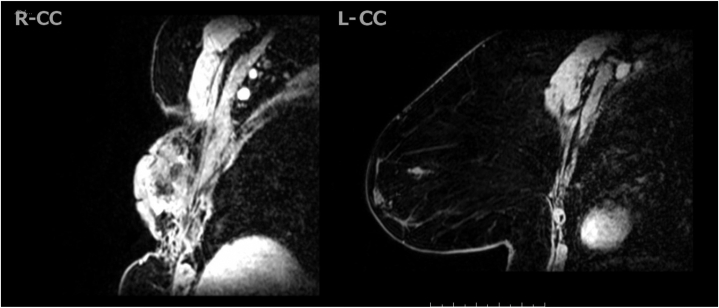
MRI of the right breast reveals contraction, skin thickening, significant abnormal enhancement, and area of probable internal necrosis spanning approximately 7.4 cm. Enhancement of the right breast extends from the skin surface to the pectoralis muscle and laterally toward the latissimus dorsi. The left breast appears unremarkable. *MRI*, Magnetic resonance imaging.

Magnetic resonance imaging of the right breast revealed severe contraction, skin thickening, significant abnormal enhancement, and area of probable internal necrosis spanning approximately 7.4 cm (Fig 3). Enhancement extended from the skin surface to the pectoralis muscle and laterally toward the latissimus dorsi.

**Fig 4:**
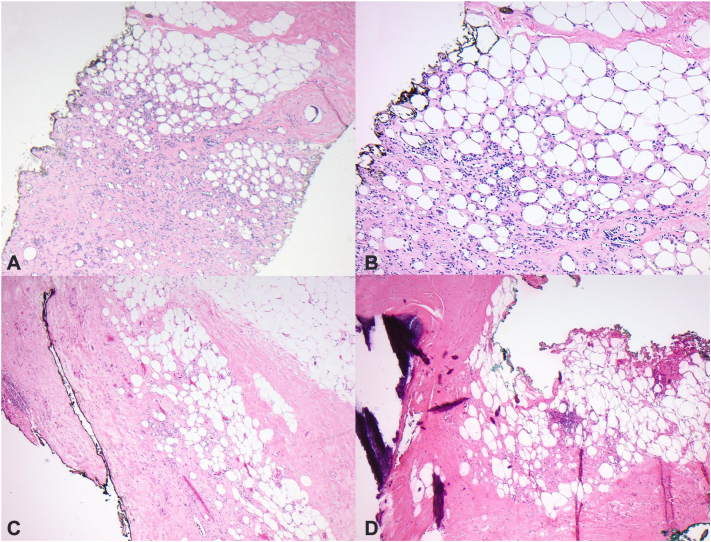
**A,** Lobular panniculitis with an infiltrate composed mainly of foamy histiocytes and lymphocytes. Fat necrosis is present. (×40) **(B)** Dense lobular infiltrate containing numerous foamy histiocytes, multinucleated giant cells, lymphocytes, and sparse plasma cells. (×100) **(C)** Subcutaneous tissue septa show marked thickening and sclerosis. There are focal lobular aggregates of chronic inflammatory cells. (×100) **(D)** Extensive subcutaneous fat necrosis, thickened fibrous septa, and calcifications. (×100) Fat lobules contain necrotic adipocytes, dystrophic calcification, and focal aggregates of chronic inflammation.

Microscopic examination revealed an atrophic and otherwise normal epidermis. Dilated vessels were present in the superficial dermis with a sparse perivascular and interstitial infiltrate of atypical fibroblasts. The subcutaneous fat showed a predominantly lobular panniculitis with thickened fibrous septa and a chronic inflammatory infiltrate composed of foamy histiocytes, multinucleated giant cells, and lymphocytes (Fig 4, *A* and *B*). Subcutaneous tissue septa were markedly thickened and sclerotic with homogenous eosinophilic collagen bundles and necrotic adipocytes at the center of the fat lobule (Fig 4, *C*). Several lobular vessels showed fibrosis of vessels walls and rare mural thrombosis. Fat lobules contained necrotic adipocytes, dystrophic calcification, and a chronic inflammatory infiltrate (Fig 4, *D*).

Immunohistochemical analysis revealed a preserved CD4:CD8 ratio. CD20 highlighted rare B cells. TIA-1 and granzyme were only focally positive. Ki-67 staining did not demonstrate a highly proliferative process. P63 and AE1/3 highlighted epithelial structures and failed to detect carcinoma. CD5 highlighted scattered T cells. CK7 highlighted rare scattered eccrine structures. CK5 highlighted epithelial and rare eccrine structures. CK8/18 was largely negative. B and T-cell gene rearrangement studies by polymerase chain reaction showed no evidence of monoclonality. Special stains for infectious organisms were negative.

The diagnosis of postirradiation pseudosclerodermatous panniculitis was made based on characteristic clinical and histopathologic findings. The patient was started on oral prednisone at 0.5 mg/kg and methotrexate (initial dose of 5 mg). At 1 month follow up, clinical disease remained active and prednisone was increased to 1 mg/kg. Over the next 6 months, the patient experienced continued progression of disease on prednisone of 1 mg/kg daily and methotrexate 15 mg weekly. Physical exam showed progression of induration to sites outside to her radiation site including the soft tissue of the lateral chest wall and the right upper abdomen. Repeat punch biopsy demonstrated continued disease activity. The patient has been tapered off prednisone and remains on methotrexate. Treatment with intravenous immunoglobulin has been initiated at doses of 2 g/kg delivered over 5 days, monthly. She continues to experience slow and asymptomatic progression of induration following 3 months of intravenous immunoglobulin infusions.

## Discussion

Post-irradiation PIPP is a rare complication of external beam radiotherapy. PIPP was first described by Winkelmann et al in 1993 in 4 women who developed induration of the breast following radiation for breast cancer, with unique histologic features including fat necrosis, lobular panniculitis, and degenerative fibrous changes.^1^ Since then, 14 additional cases have been reported in the literature.^1^, ^2^, ^3^, ^4^, ^5^, ^6^, ^7^, ^8^, ^9^ The most common involved area is the anterior chest wall following radiotherapy for breast cancer. While most reported cases are localized to the subcutis of the radiation field, our case uniquely involved the entirety of breast parenchyma and subcutaneous tissue extending to the thoracic muscles.

The differential diagnosis for our case included recurrent carcinoma, deep morphea, lupus panniculitis, radiation fibrosis, and subcutaneous panniculitis-like T-cell lymphoma. Histopathologically, PIPP has characteristic findings including a mostly lobular panniculitis with fat necrosis and a chronic inflammatory infiltrate composed mainly of foamy histiocytes and lymphocytes.^1^, ^2^, ^3^, ^4^, ^5^, ^6^ Thickening and sclerosis of the connective tissue septa is also classic. In contrast, deep morphea is mostly a septal panniculitis containing lymphocytes and plasma cells at the interface between fat lobules and connective tissue septa. Radiation fibrosis demonstrates extensive homogenous sclerosis of the subcutaneous tissue in the absence of a lobular panniculitis. In contrast to PIPP, the infiltrate of lupus panniculitis is lymphocyte-predominant and adipocyte necrosis would not be expected. Recurrent carcinoma was excluded by the absence of mammary ductal epithelial cells on consecutive core biopsies and subcutaneous T cell lymphoma was excluded by immunohistochemistry and B- and T-cell gene rearrangement studies.

In addition to her dramatic clinical presentation, our patient’s case occurred 27 years following radiotherapy, making this case the longest latency period between radiation and onset of PIPP to date. Most patients present in the first year following treatment and few cases have been delayed by 1-5 years.^1^, ^2^, ^3^, ^4^, ^5^, ^6^ Previously, the longest latency period reported was 17 years.^4^

Due to its rarity, the course of PIPP is not well characterized and its response to treatment has not been established. Our case is the first to describe an attempted treatment course, and our patient unfortunately experienced disease progression despite a 6-month course of high-dose oral prednisone. Our selection to trial intravenous immunoglobulin next was made in part by its observed immunomodulatory and antifibrotic activity in other sclerosing skin conditions such as generalized morphea and systemic sclerosis.^10^

While our case is the first to describe resistance to therapy, several cases of localized PIPP in the literature have reported spontaneous remission with or without the use of topical steroids. In the 4 cases originally described by Winkelmann et al, all 4 patients displayed limited subcutaneous disease and showed improvement without intervention or with the use of topical steroids within 2-5 m.^1^ In 2001, Carrasco et al described 4 more females who developed localized PIPP following radiation that showed progressive improvement over 1-4 years without any treatment, suggesting the disease may burn out on its own in time.^2^ In contrast to these reports, our patient exhibited diffuse subcutaneous and parenchymal involvement and failed to respond to oral steroids at a maximum dose of 1 mg/kg. To date, there are no known therapies to treat PIPP or induce its remission.

## Conflicts of interest

None disclosed.
